# An ERβ agonist induces browning of subcutaneous abdominal fat pad in obese female mice

**DOI:** 10.1038/srep38579

**Published:** 2016-12-06

**Authors:** Yi-fei Miao, Wen Su, Yu-bing Dai, Wan-fu Wu, Bo Huang, Rodrigo P. A. Barros, Hao Nguyen, Laure Maneix, You-fei Guan, Margaret Warner, Jan-Åke Gustafsson

**Affiliations:** 1 Center for Nuclear Receptors and Cell Signaling, Department of Biology and Biochemistry, University of Houston, Houston, TX 77204, USA; 2 Center for Nephrology and Urology, Department of Physiology, Shenzhen University Health Science Center, Shenzhen University, Shenzhen, 518060, China; 3 Advanced Institute for Medical Sciences, Dalian Medical University, Dalian, 116044, China; 4 Center for Innovative Medicine, Department of Biosciences and Nutrition, Karolinska Institutet, Novum, 14186 Stockholm, Sweden

## Abstract

Estrogen, via estrogen receptor alpha (ERα), exerts several beneficial effects on metabolism and energy homeostasis by controlling size, enzymatic activity and hormonal content of adipose tissue. The actions of estrogen on sympathetic ganglia, which are key players in the browning process, are less well known. In the present study we show that ERβ influences browning of subcutaneous adipose tissue (SAT) via its actions both on sympathetic ganglia and on the SAT itself. A 3-day-treatment with a selective ERβ agonist, LY3201, induced browning of SAT in 1-year-old obese WT and ERα^−/−^ female mice. Browning was associated with increased expression of ERβ in the nuclei of neurons in the sympathetic ganglia, increase in tyrosine hydroxylase in both nerve terminals in the SAT and sympathetic ganglia neurons and an increase of β3-adrenoceptor in the SAT. LY3201 had no effect on browning in young female or male mice. In the case of young females browning was already maximal while in males there was very little expression of ERβ in the SAT and very little expression of the β3-adrenoceptor. The increase in both sympathetic tone and responsiveness of adipocytes to catecholamines reveals a novel role for ERβ in controlling browning of adipose tissue.

Obesity is due to the chronic imbalance between energy intake and energy expenditure^1^. The tendency of postmenopausal women to gain weight suggests a role for estrogen in controlling white adipose tissue (WAT)^2^,^3^. The role of estrogen in regulating obesity has been well documented in mice. Both ERα^−/−^ mice^4^,^5^ and aromatase^−/−^ mice^6^,^7^ become obese. Many of the metabolic effects of estrogen are mediated by ERα and its deficiency in both male and female mice results in body weight gain and adiposity predominantly through reducing energy expenditure^5^,^8^. In addition, increased ERα signaling suppresses energy intake and induces energy expenditure^9^,^10^. Some of the effects of estrogen on body fat are mediated by the activation of ERα in the ventral medial nucleus of the hypothalamus (VMH)^8^,^11^. Estradiol (E2) has been reported to increase energy expenditure through ERα and activation of sympathetic nervous system (SNS)-brown adipose tissue (BAT) axis^12^. ERβ, on the other hand, has direct anti-lipogenic and anti-adipogenic effects in adipocytes as a negative regulator of peroxisome proliferator-activated receptor γ (PPARγ)^13^. In addition, selective ERβ agonists show PPARγ antagonistic actions in adipocytes and reduce body weight and adipose tissue composition in mice fed a high-fat diet or in ovariectomy-induced obesity^14^.

BAT which is specialized for the dissipation of chemical energy in the form of heat, is able to protect mammals against hypothermia^15^, obesity^16^ and type 2 diabetes^17^. Unlike rodents which have BAT throughout life, adult humans harbor distinct depots of cold-inducible brown adipocytes which are dispersed within WAT in the supraclavicular, para-aortic and suprarenal regions^18^,^19^,^20^. Adipose tissues undergo browning following cold exposure^19^ and this is mediated by an increase in sympathetic tone. The possibility that induction of browning of WAT could be used to combat obesity is of potential clinical interest.

There is no direct evidence that ERβ influences browning of WAT through its actions in either the central or peripheral nervous system. In the present study, we used the abdominal subcutaneous fat pad as our source of SAT. The subcutaneous abdominal fat pad in females houses the mammary gland. We found that short-term activation of ERβ with a specific agonist, LY3201, significantly induced browning in this fat pad through increased sympathetic tone in the thoraco-lumbar sympathetic ganglia as well as direct actions on the SAT where it induced expression of the β3-adrenoceptor.

## Results

### Activation of ERβ induces browning of mammary white adipose tissue in 1-year-old female mice

**Figure 1:**
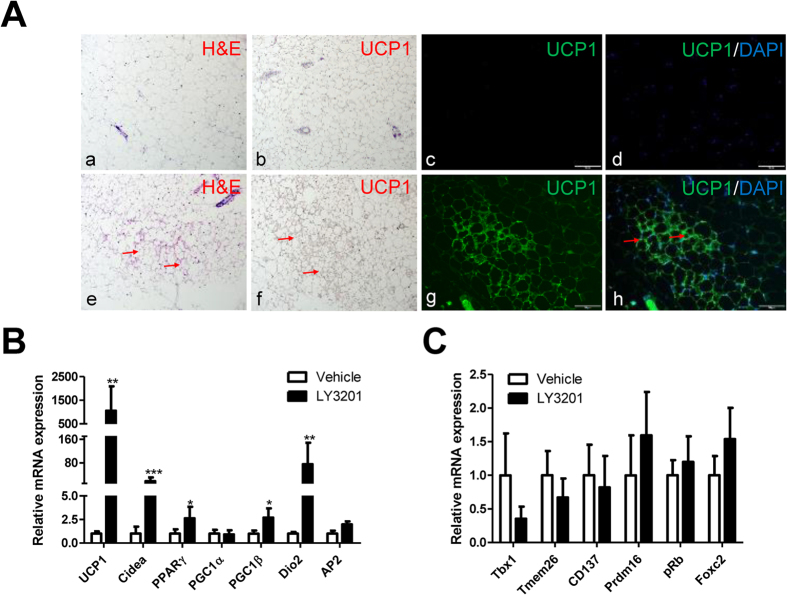
Increased browning of SAT in obese WT female mice after treatment with LY3201 for 3 days. (**A**) Treatment with LY3201 induced multilocular lipid droplets, as shown by H&E staining (red arrow) in e, and UCP1 positive (red arrow) as represented in f–h. All pictures are 20x magnification. (**B**) There was up-regulation of browning-associated genes by LY3201 in SAT of aged WT female mice. (**C**) There were no changes in beige-cell-specific genes after treatment with LY3201. *p < 0.05, **p < 0.01, ***p < 0.001, LY3201 vs. Vehicle.

1–year-old female wild type (WT) and ERα^−/−^ mice were used because they develop late-onset obesity. Treatment with the ERβ agonist, LY3201, for three days markedly increased multilocular lipid-droplets (Fig. 1Aa and e) and increased the number of UCP1-positive adipocytes in female SAT as shown in Fig. 1Ab–d and f–h. This short-term treatment had no effect on total body weight or the ratio of the liver, mammary fat pad, BAT or gonadal WAT to body weight (Supplementary Fig. S1A–F). Consistent with the morphological changes, qPCR results confirmed that the thermogenic genes such as *UCP1*, *Cidea*, *PPAR*γ, *PGC1*β and *DIO2* were significantly increased in the mammary fat pad of LY3201-treated group (Fig. 1B). However, there was no induction of *AP2* (Fig. 1B), indicating that the browning process was not due to adipogenesis. This lack of adipogenesis was also supported by qPCR analysis showing a lack of change in proliferative genes, such as *cyclin A2*, *cyclin B1*, *PCNA*, *Ki67*, *C/EBP*α and *C/EBPδ* (Supplementary Fig. S1-G). Despite the induction of genes involved in browning of white adipose tissue, none of the markers of beige cells (*Tbx1*, *Tmem26*, *CD137*, *Prdm16*, *pRB* and *Foxc2*) were altered in SAT by LY3201 (Fig. 1C). Thus beige cells do not appear to be involved in ERβ-ligand-induced browning.

### Short-term activation of ERβ does not evoke the browning of SAT in young female or male mice

**Figure 2:**
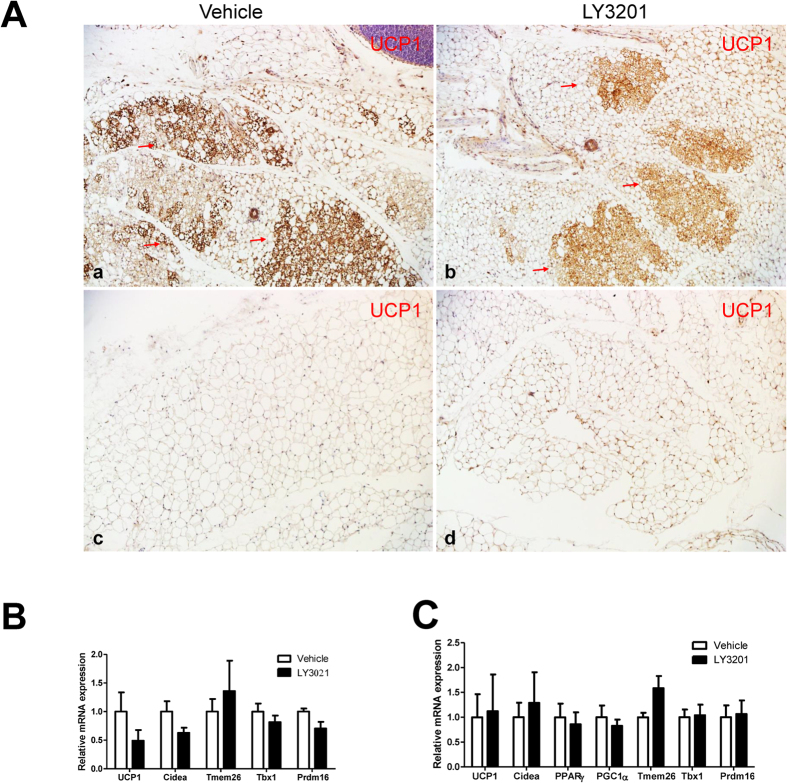
Effects of 3-day treatment with LY3201 on 3-month-old female and male. (**A**) UCP1 staining (red arrows) in abdominal SAT of female (a,b) or SAT of male mice (c,d). Red arrows indicate adipocytes with multilocular lipid-droplets. All pictures are 20x magnification. In line with the lack of morphological changes, LY3201 did not affect the level of mRNA of BAT-expressed genes in female (**B**), male (**C**).

**Figure 3:**
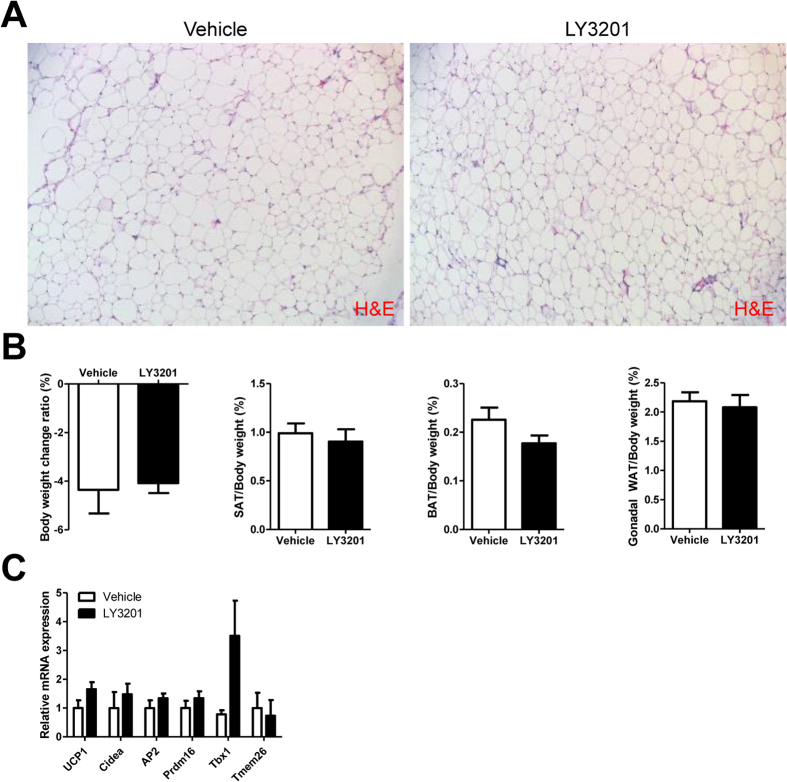
Effect of LY3201 in SAT of 12-month-old male obese WT mice. (**A**) LY3201 did not change the size or morphology of adipocytes in SAT as shown by H&E staining. All pictures are 20x magnification. There was no effect on body weight, adipose tissue organ weight (**B**) or browning or beige cell related genes (**C**).

After a 3-day exposure to LY3201, no significant increase in browning was observed as measured by a lack of change in expression of either UCP1 protein (Fig. 2Aa–d) or the mRNA level of browning-related genes (Fig. 2B and C) in 3-month-old female or male SAT. Of note, in young females (Fig. 2Aa), there are much more BAT in the SAT than in 1-year-old females (Fig. 1Aa) or in males (Fig. 2Ac). When LY3201 was administered to 1-year-old male WT mice, there was no browning of SAT, as shown by the unaltered adipocyte morphology (Fig. 3A). No changes in body weight, organ weights (Fig. 3B) or browning-related genes were observed (Fig. 3C).

### Expression of ERβ in SAT

**Figure 4:**
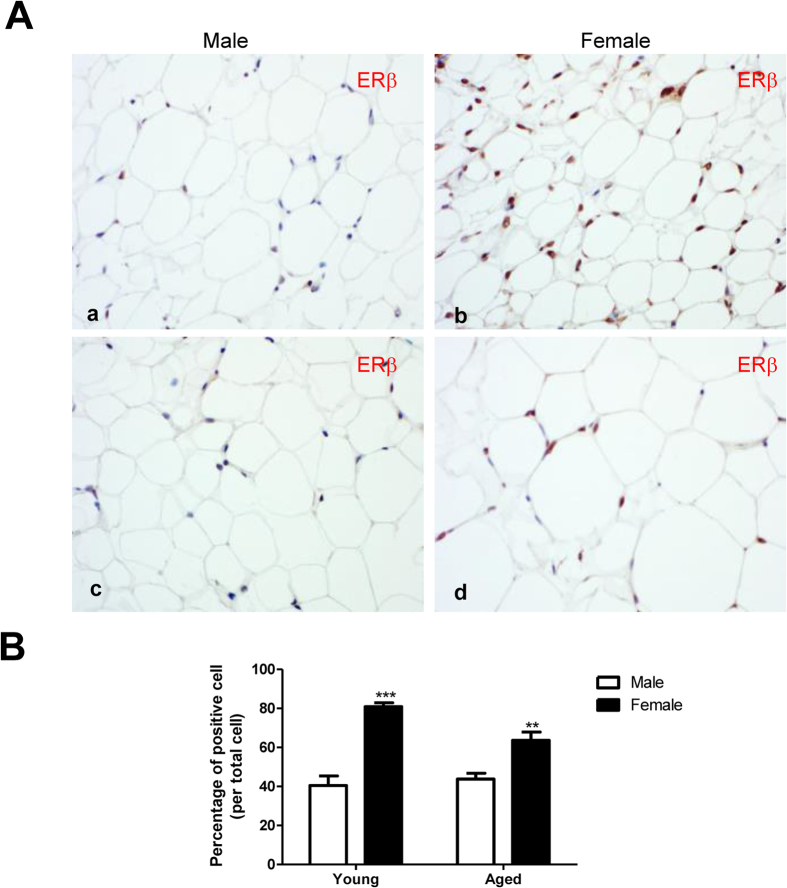
Expression of ERβ in SAT from 3- and 12-month-old male and female mice. (**A**) Expression of ERβ in the nuclei of SAT in young and aged male WT mice was similar (a,c). Expression of ERβ in the nuclei of SAT in 3- and 12-month-old female WT mice was higher than in age-matched male mice (b,d). (**B**) Quantification of ERβ. Higher expression of ERβ was observed in female SAT than in male SAT. **p < 0.01, ***p < 0.001, female vs. male. All pictures are 40x magnification.

Expression of ERβ was high in the nuclei of adipocytes in 3-month-old female SAT (Fig. 4Ab) and there was a decrease in expression of ERβ in SAT of 1-year-old females (Fig. 4A and B). In males, very few nuclei in the SAT were ERβ-positive (Fig. 4Aa and c, Fig. 4B). Thus the lack of response to LY3201 in males appears to be due to low expression of ERβ in the SAT while in 3-month-old-females it is due to an already fully activated ERβ.

### Activation of ERβ induces browning of SAT in 1-year-old ERα^−/−^ female mice but not ERβ^−/−^ female mice

**Figure 5:**
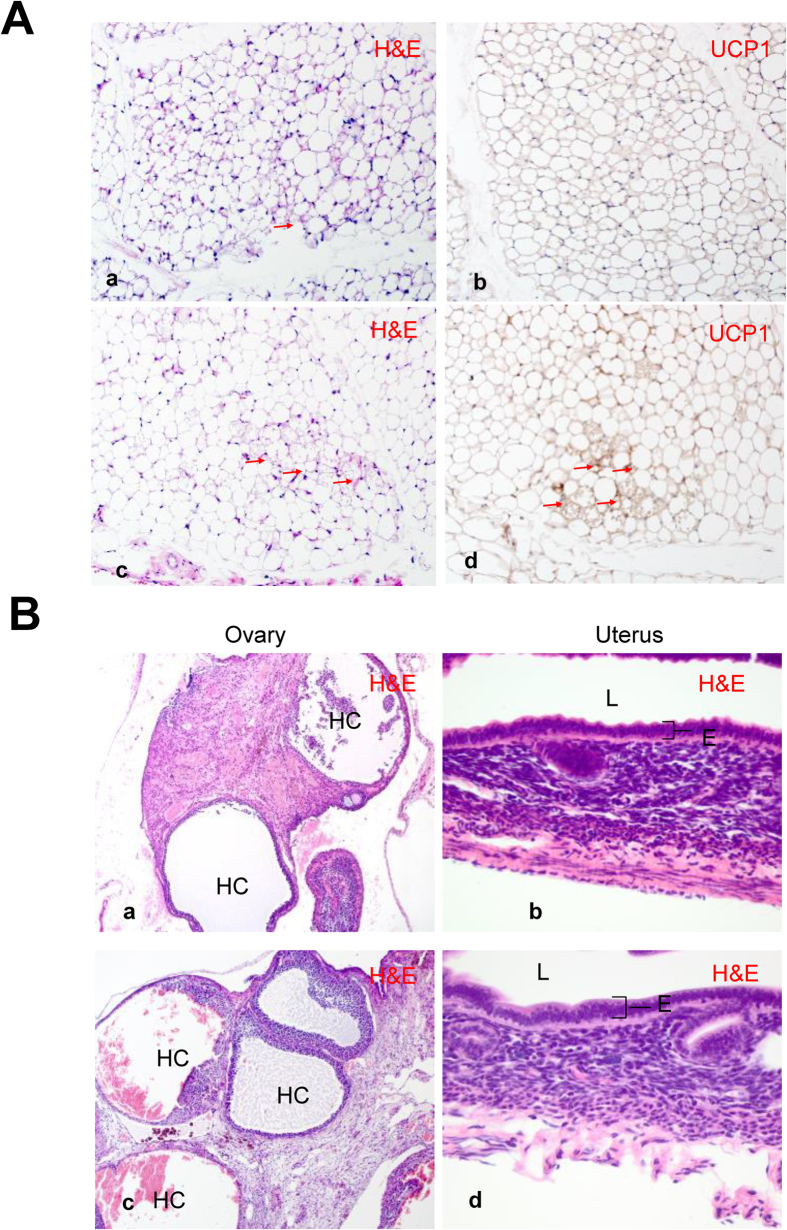
Significant browning of abdominal SAT in aged, obese female ERα^−/−^ mice. (**A**) Treatment with LY3201 induced more multilocular lipid droplets as indicated by H&E staining (red arrow) (a,c) and expression of UCP1 (red arrow) (b,d). All pictures were 20x magnification. (**B**) LY3201 did not affect ovary or uterus morphology in female ERα^−/−^ mice. As expected there were many hemorrhagic cystic follicles (HC) and lack of mature follicles and corpora lutea in ERα^−/−^mice. ERα^−/−^ mice uterus was also hypoplastic with thinner epithelial layer (b,d). However, treatment with LY3201 did not further change the morphology of ovaries (a,c) or uterus (b,d). E: Epithelial layer, L: lumen of the organ. Pictures of ovaries are 10x magnification. Pictures of uterus are 20x magnification.

In order to rule out the possibility that the ERβ-selective agonist was acting through ERα, we treated 1-year-old ERα^−/−^ female mice with LY3201 for 3 days. The mice were confirmed to be ERα^−/−^ by genotyping and morphological abnormalities in the ovary and uterus indicative of disruption of ERα (markedly atrophic uteri and hemorrhagic cystic ovaries). Treatment with LY3201 induced browning of SAT as illustrated by H&E staining and UCP1 immunohistochemistry (Fig. 5A) but did not correct the abnormalities in the uterus and ovary of ERα^−/−^mice (Fig. 5B). To further confirm that the browning effect of LY3201 was mediated by ERβ, we examined the SAT of ERβ^−/−^ mice and its response to LY3201. There was a marked absence of BAT in the SAT of ERβ^−/−^ mice and LY3201 treatment failed to induce browning (Supplementary Figure 2). These findings suggest that LY3201 is an effective ERβ-selective agonist, and activation of ERβ is responsible for LY3201-induced browning in the SAT.

### Effects of LY3201 on the sympathetic nervous system (SNS)

**Figure 6:**
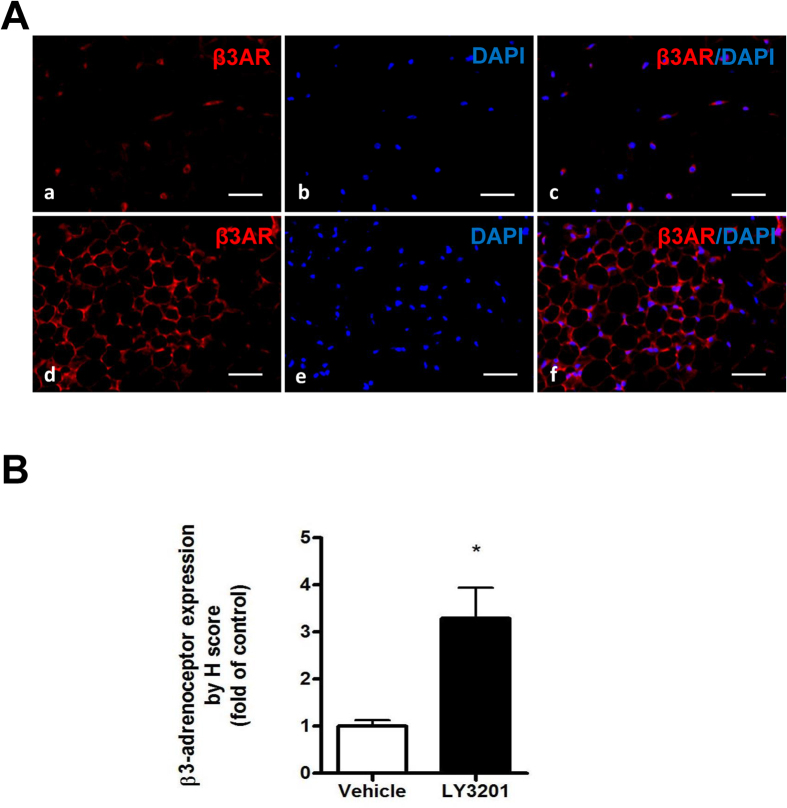
Expression of β3-adrenoceptor in SAT from 1-year-old obese WT female mice treated with ERβ agonist LY3201. (**A**) β3-adrenoceptor (β3AR, Red) immunofluorescence in both vehicle and LY3201 treated SAT. (Blue, DAPI) (**B**) β3-adrenoceptor quantification in vehicle and LY3201 treated SAT. **p < 0.01, LY3201 vs. Vehicle. All pictures are 40x magnification.

In addition to direct effects on the adipose tissue itself, both BAT and the browning of white adipocytes are also controlled by SNS^21^,^22^,^23^. It has been reported that intra-hypothalamic estrogen injection regulates SNS outflow and estradiol inhibits AMPK through ERα in VMH, leading to the activation of thermogenesis in BAT through SNS^12^. Norepinephrine (NE) released by sympathetic nerve terminals in SAT activates β3-adrenoceptor, a key factor in the browning process^24^. We examined the expression β3-adrenoceptor in SAT. As shown in Fig. 6A and B, LY3201 treatment strongly induced β3-adrenoceptor expression in 1-year-old female SAT.

**Figure 7:**
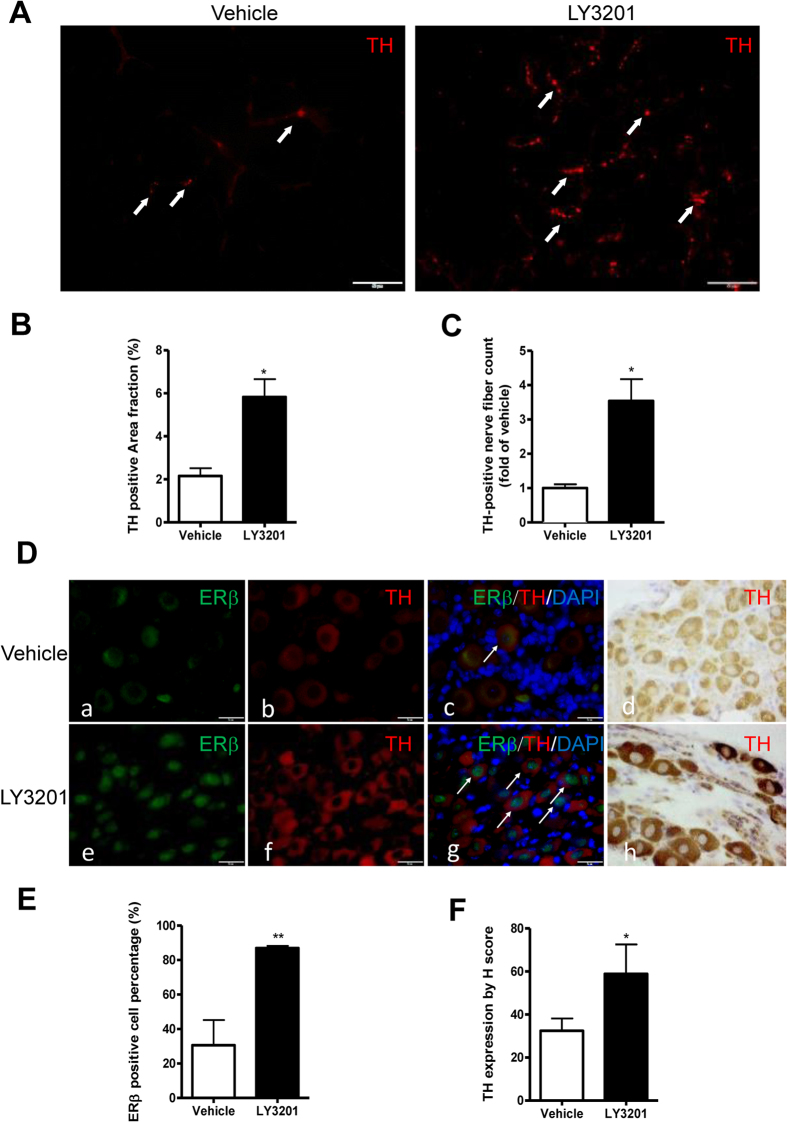
Expression of TH in SAT and sympathetic ganglia from 1-year-old obese WT female mice treated with LY3201. (**A**) TH expression in SAT was significantly increased after LY3201 treatment. Arrowheads indicate TH-positive fibers. TH-positive areas and TH-positive nerve fiber count in SAT in Vehicle and LY3201-treated mice were quantified (**B**,**C**). LY3201 causes a marked increase in TH expression in the SAT. (**D**) Immunostaining of ERβ (green) and TH (red) in thoraco-lumbar sympathetic neurons with or without LY3201 treatment (a,e,b,f). Both immunofluorescence (b,f) and immunochemistry (d,h) indicate that TH expression was strongly induced in sympathetic neurons after LY3201 treatment. Merged images of ERβ and TH (c,g). The white arrows indicate positive nuclear staining of ERβ. (**E**) Quantification of ERβ positive cell percentage in vehicle and LY3201-treated sympathetic ganglia. (**F**) TH quantification in vehicle and LY3201-treated sympathetic ganglia. All pictures are 100x magnification. *p < 0.05, LY3201 vs. Vehicle.

Tyrosine hydroxylase (TH) is the initial and rate limiting step in NE bio-synthesis. It has been reported that WAT browning is positively correlated with parenchymal TH-positive nerve fiber density^22^. Thus, we hypothesized that LY3201 was acting on the peripheral sympathetic nervous system to increase TH expression in nerve terminals on the surface of adipocytes in the SAT. Treatment with LY3201 strongly induced expression of TH in nerve terminals in the adipocytes of SAT in 1-year-old WT female mice (Fig. 7A and B). To determine whether this increase was due to changes in the sympathetic ganglia, mouse thoraco-lumbar sympathetic ganglia were excised, fixed and paraffin embedded. Immunohistochemical staining of tyrosine hydroxylase was used to visualize catecholaminergic neurons of the sympathetic ganglia. We found that in 3-month-old female mice, ERβ was present in the nuclei of 90% of sympathetic ganglia (Supplementary Fig. S3) but in 1-year-old female mice, nuclear ERβ expression was markedly reduced so that only 30% of neuronal nuclei were positive (Fig. 7Da and E) and instead ERβ was found in the cytoplasm.

**Figure 8:**
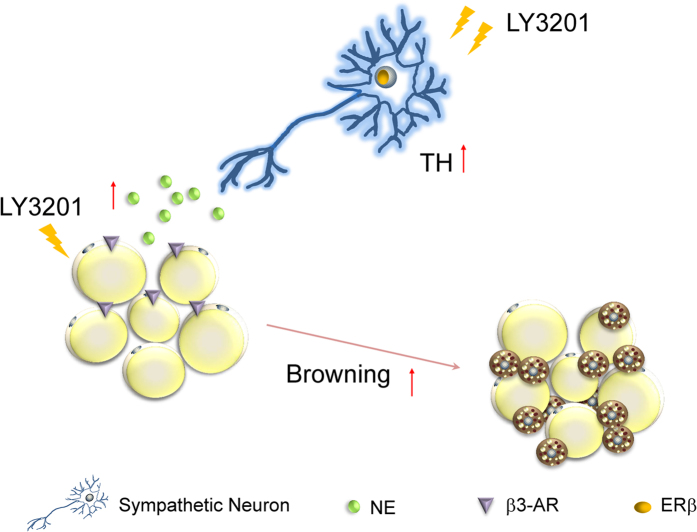
Schematic diagram of actions of ERβ in controlling TH expression in the sympathetic ganglia and browning of abdominal SAT in female mice with late-onset obesity. In female mice, with late-onset obesity there is reduced ERβ expression in the nucleus of sympathetic neurons, and short term-treatment with LY3201 increased ERβ expression. LY3201 was able to up-regulate expression of TH in sympathetic neurons. Through activating the synthesis of TH, and stimulation of the expression β3-adrenoceptor LY3201 leads to the browning of SAT.

Treatment with LY3201 caused a marked up regulation of both nuclear ERβ (2.9-fold change) and cytoplasmic TH (1.8-fold change) (Fig. 7D, E and F). After treatment with LY3201, 87% of nuclei in the neurons were ERβ-positive in 1-year-old females (Fig. 7D and E, Fig. 8). ERα expression was not changed after ERβ agonist treatment (Supplementary Fig. S4).

### Reason for lack of response of males

To address the question of why LY3201 had no effect on male mice, we examined ERβ expression pattern in male sympathetic ganglia. Unlike the females, in 1-year-old males there was robust expression ERβ in the nuclei of the neurons. (Supplementary Fig. S5a and b). Thus lack of response of males to LY3201 is not due to loss of ERβ in the nuclei of sympathetic neurons. There was also a gender difference in the neurotransmitters with which ERβ was co-localized. In females, ERβ in sympathetic ganglia was mainly expressed in TH positive neurons, while in males it was more widely expressed in NYP-, VIP- and CGRP-positive neurons (Supplementary Fig. S5c–h).

The reason for the lack of response to ERβ agonist in males appears to be the relatively low expression level of ERβ in male SAT in both young and aged groups (Fig. 5A and B). As a consequence of this, ERβ ligands failed to induce β3-adrenoceptor in SAT (Supplementary Fig. 6). The low expression of ERβ in male SAT has been reported by others^25^ but the reason for this gender difference remains to be investigated.

## Discussion

Previous studies have indicated that ERβ exerts a beneficial effect on energy metabolism^9^,^26^. Long-term treatment with an ERβ agonist did not only ameliorate diet-induced obesity in both male and female rodents, but also improved metabolic outcome in the animal model of postmenopausal obesity^14^,^27^. It is well-known that ERα predominantly mediates increased energy expenditure induced by estrogen through ventromedial nucleus of the hypothalamus (VMH)^8^,^12^. Here, we have demonstrated the importance of ERβ in regulating browning in the abdominal WAT fat pad through its effects both on the sympathetic nervous system and on the WAT itself.

The ERβ selective agonist, LY3201, had no detectable effects on the subcutaneous white adipose tissue in either males or 3-month-old females. The reason for this observation may be a combination effect of gender difference in both sympathetic ganglia and SAT. First, there is robust expression of ERβ in the nuclei of the neurons of sympathetic ganglia in young females and males so the receptor appears to be fully active. In 1-year-old females, ERβ is mostly cytoplasmic in these neurons and a ligand is required for shifting the receptor into the nucleus. The reason for the nuclear localization of ERβ in young females is most likely the high estrogen levels in these mice. The maintenance of ERβ in the nuclei in males needs further investigation but it may be related to high levels of 5α-androstane-3β, 17β-diol, which is an ERβ physiological ligand synthesized from 5α-dihydrotestosterone (DHT) in males^28^.

The second factor is the regulation of the β3-adrenoceptor in SAT by the ERβ agonist. Male SAT expresses low levels of ERβ and expression of the β3-adrenoceptor in their SAT was as low as that in 1-year-old females. Thus the male SAT is less sensitive to adrenergic stimulation. In human mature white adipocytes, ERβ mRNA levels are higher in women than in men in both intra-abdominal and subcutaneous regions^25^ and 17β-estradiol specifically upregulates the expression of ERβ mRNA in SAT from women but not from men^25^. The reason for this lack of ligand-induced increase in ERβ expression in male SAT remains to be examined. In other organs such as brain, LY3201 increases the expression of ERβ in male mice^29^.

ERβ is a master repressor of PPARγ in the visceral white adipocytes, either through indirect antagonistic actions and abrogation of the ability of PGC-1 to co-activate, or through direct inhibition of its transcriptional activity^13^. We found that in the subcutaneous white adipose tissue depots, activation of ERβ did not augment adipogenesis or proliferation, since neither *AP2* (signature adipogenic marker) or proliferative genes were up-regulated. LY3201 had small effects on *PPAR*γ and *PGC-1*β and this suggests that the regulation of these two genes is more complex than simply activation by ERβ.

Beige cells largely account for the browning of WAT in the male rodents upon exposure to cold^30^. However, in the present study despite the robust browning induced by LY3201 in the female mammary fat pad, there was no evidence of beige cells. Clearly there are gender differences in the abdominal subcutaneous fat pad. In addition to the white-brown transformation under cold-stimulation, the female mammary adipocytes have the capacity to trans-differentiate into mammary glandular tissue during pregnancy and lactation^31^,^32^,^33^. One of the gender differences in this fat pad is the high expression of ERβ in the females. In the present study, the 3-day exposure to LY3201 caused no obvious increase in the mammary epithelium.

ERα is the predominant estrogen receptor in the interscapular BAT and mediates most of the changes in energy metabolism evoked by estrogen^12^. Estrogen through ERα regulates brain-derived neurotrophic factor (BDNF) in the hypothalamus which in turn has been linked to selective SNS modulation of white fat to induce browning^34^,^35^. Central administration of estrogen, acting through VMH, activates SNS and increases thermogenesis^12^. In this study, we report that the ERβ selective ligand LY3201 can induce tyrosine hydroxylase in the thoraco-lumbar sympathetic ganglia and their terminals on SAT and induce browning in SAT.

TH is transcriptionally regulated by estradiol both *in vivo* and *in vitro*^20^,^21^,^22^,^23^. Marwarha *et al*. reported that the binding capacity of ERβ to the ERE in the TH promoter is about 10-fold greater than the binding of ERα to the ERE and several groups have reported half ERE and AP1 sites in the TH promoter region^36^. The non-classical pathway (tethering to AP1 sites) is the preferred mode of action of ERβ. We found that the ERβ-specific agonist, LY3201, markedly increased (2.9-fold change) number of ERβ-positive nuclei in sympathetic neurons. These results demonstrate the specific roles of ERβ in regulation of TH expression.

Collectively, the present work has provided evidence that ERβ is essential for maintaining sympathetic tone in thoraco-lumbar sympathetic ganglia of aging female mice. The role of ERβ in induction of the β3-adrenoceptor in SAT is a novel finding and is further evidence of the gender differences in this adipose tissue. The function of the BAT in the mammary fat pad is not clear and needs to be further investigated.

## Materials and Methods

### Animals

Twelve-week-old male and female C57BL/6 WT, 1-year-old male and female C57BL/6 WT and ERα^−/−^ mice, were housed under standard conditions and ambient temperature (23 °C) with free access to water and chow diet throughout the study. At the end of each study, mice were deeply euthanized with CO_2_ and tissues were quickly harvested, weighed and fixed or frozen for further study. All experimental protocols were approved by the Institutional Animal Care and Use Committee at the University of Houston, and adhered to the National Institutes of Health Guidelines for the Care and Use of Laboratory Animals. Effort was made to minimize the number of animals used and their suffering.

### *In vivo* treatment with ERβ agonist

The ERβ agonist LY3201, (3aS, 4R, 9bR)-2, 2-difluoro-4-(4-hydroxyphenyl)-3, 3a, 4, 9b-tetrahydro-1H-cyclopenta[c] chromen-8-ol (CAS 787621-78-7), was a gift from Eli Lilly^37^. Mice were randomly separated into vehicle- or LY3201-treated groups. Pellets of LY3201 were prepared by Innovative Research of America (Sarasota, FL) to release (0.04 mg/d). They were implanted on the lateral side of the neck between the ear and the shoulder. The pellet is made of a matrix fused with an active product. The ingredients are: cholesterol, cellulose, lactose, phosphates, and stearates. After continuous release of the pellet for 3 days, all mice were anesthetized deeply with CO_2_.

### Gene expression analysis

50 mg–100 mg SAT were used for total RNA extraction by using Qiagen RNeasy lipid tissue mini kit following manufacturer’s protocol. The lymph node within SAT was removed before immersing the tissue in Trizol. cDNA was synthesized using random hexamer primer and revertAid first strand cDNA synthesis kit (Invitrogen). Quantitative PCR (qPCR) was performed using iTaq universal SYBR green supermix (Bio-rad) and primers applied are listed in Supplementary Table S1.

### Immunohistochemistry

All mice were transcardially perfused with heparinized saline followed by 4% (wt/vol) paraformaldehyde in 0.1M PBS (pH = 7.4). Sympathetic ganglia (SG), SAT, ovary and uterus were dissected and post-fixed in the same fixative solution overnight at 4 °C. After fixation, tissues were transferred to 70% ethanol and processed for paraffin sectioning (5 μM). Paraffin sections were deparaffinized in xylene, rehydrated and then incubated with 3% H_2_O_2_ in PBS for 30 min at room temperature to quench endogenous peroxidase. Unspecific binding was blocked with incubation of 3% (wt/vol) bovine serum albumin (BSA, Sigma-Aldrich) in PBS for 1 h at room temperature. Then, these sections were subjected to immunohistochemistry staining at 4 °C overnight with first antibodies as diluted in 1% BSA respectively: UCP1 antibody (Abcam, ab10983, 1:500), TH antibody (Millipore, AB152, 1:400), ERα antibody (Abcam, ab37438, 1:200), ERβ antibody (made by our own lab, 1:200). After washing, sections were incubated with biotinylated secondary antibody (1:200 dilution) for 1 h at room temperature and then Vectastain ABC kit (Vector laboratories) was used according to manufacturer’s instruction. After washing with PBS, peroxidase activity was visualized with DAB staining (Thermo Scientific) and counterstained with Mayer’s hematoxylin (Sigma-Aldrich), dehydrated through an ethanol series to xylene and mounted with Permount (Fisher Scientific).

### Morphometric analysis

TH-positive parenchyma nerve fibers were counted. Perivascular fibers were not considered in the quantitative analysis. Ten randomly selected areas at high magnification per animal were studied using an Olympus light microscope (Olympus America Inc.) with a ×100 oil immersion objective at ×1000 final magnification. TH-positive threshold is manually set and TH-positive area fraction is calculated by software automatically. For the TH immunochemistry semi-quantification of sympathetic ganglion, an H-score system was performed as previously described^38^.

### Statistical analysis

For the quantification of positive rate of ERs and TH staining, randomly selected staining slides from three mice in each group and more than six representative fields of each slide were analyzed by National Institutes of Health (NIH) ImageJ software. The positively stained adipocyte cells were counted and scored by the percentage of the stained cells to the total adipocytes in each slide. Data were expressed as mean ± SD. Statistical comparisons were made using student’s t-test or a one-way ANOVA followed by a Newman-Keuls post-test. *p* < 0.05 was considered as significant difference.

## Additional Information

**How to cite this article:** Miao, Y.-f. *et al*. An ERβ agonist induces browning of subcutaneous abdominal fat pad in obese female mice. *Sci. Rep.* **6**, 38579; doi: 10.1038/srep38579 (2016).

**Publisher's note:** Springer Nature remains neutral with regard to jurisdictional claims in published maps and institutional affiliations.

## Supplementary Material

Supplementary Information
