# Supplementary material for: An ERβ agonist induces browning of subcutaneous abdominal fat pad in obese female mice
Source: Sci Rep. 2016 Dec 6;6:38579. doi: 10.1038/srep38579 (PMC5138613; doi:10.1038/srep38579)
Supplement: Supplementary Information [file srep38579-s1.pdf]

Supplementary Information

An ERβ agonist induces browning of subcutaneous abdominal fat pad in obese female mice

Yi-fei Miao[1, †], Wen Su[2, †], Yu-bing Dai[1], Wan-fu Wu[1], Bo Huang[1], Rodrigo P. A. Barros[1], Hao Nguyen[1], Laure Maneix[1], You-fei Guan[1,3], Margaret Warner[1] and Jan-Åke Gustafsson[1,4,*]

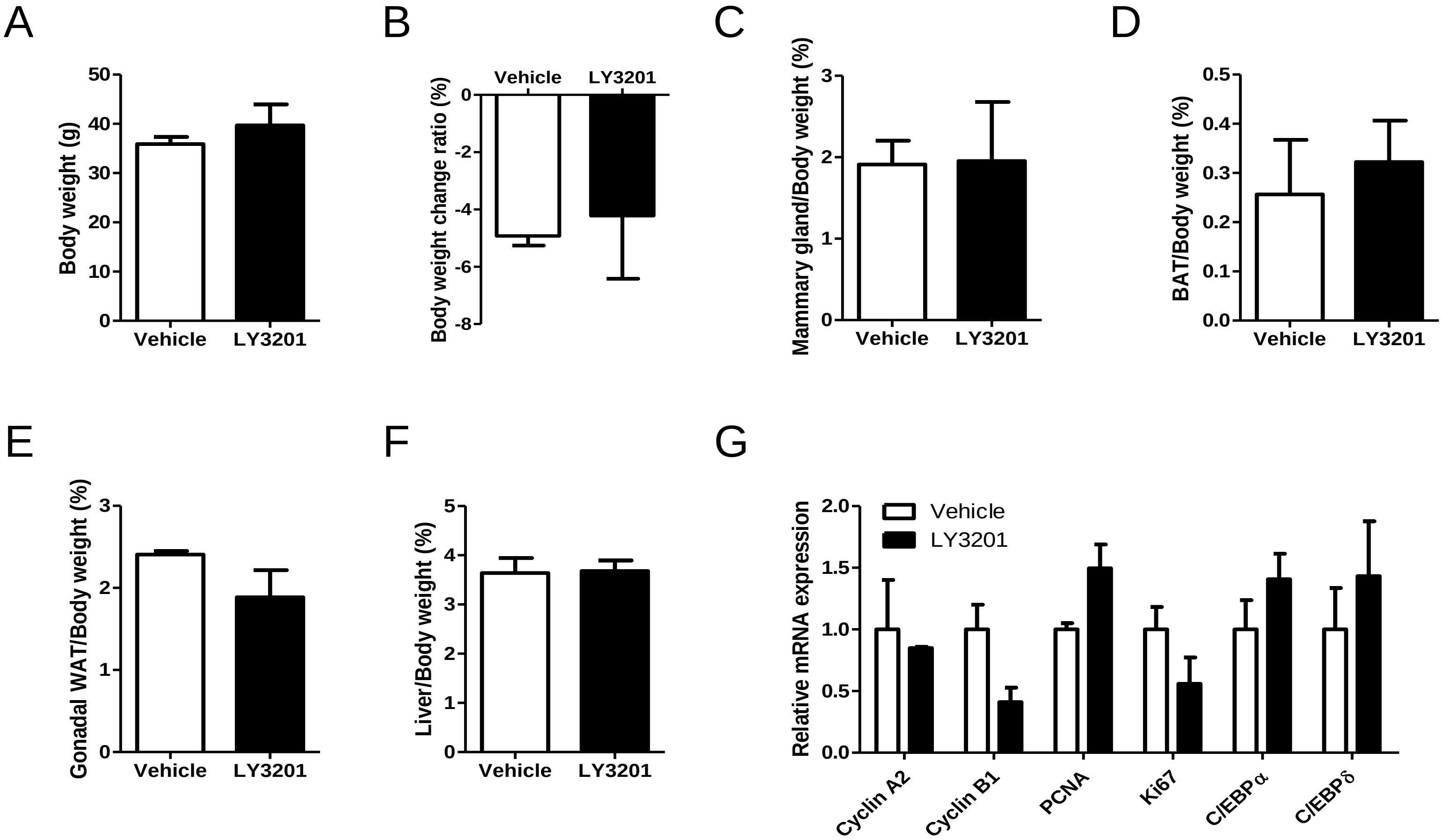

Supplementary Figure S1. No change of body weight or organ weights after the treatment with LY3201 in aged, obese female WT mice. A short term treatment with LY3201 did not change the body weight (A&B) or lipid-metabolically active organs weights (C-F). (G) ERβ ligand did not induce cell proliferative genes in MG.

Supplementary Figure S1

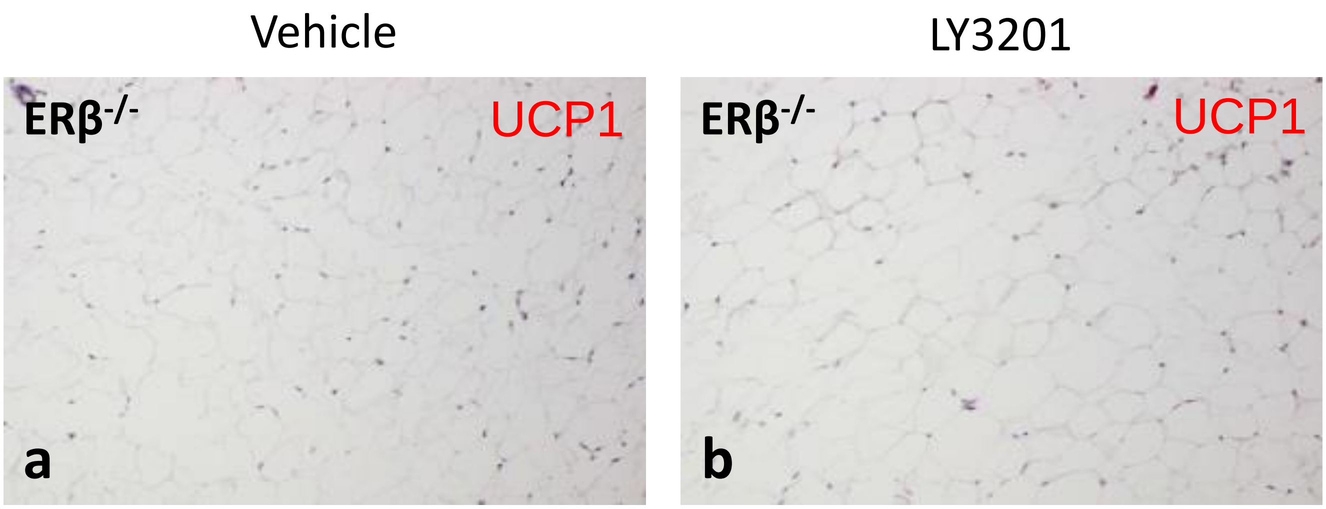

Supplementary Figure S2. Immunohistochemistry results showed that LY3201 treatment failed to induce UCP1 protein expression in 1-year-old ERβ knockout female mice (Left panel is vehicle group representative, right panel is LY treated group representative).

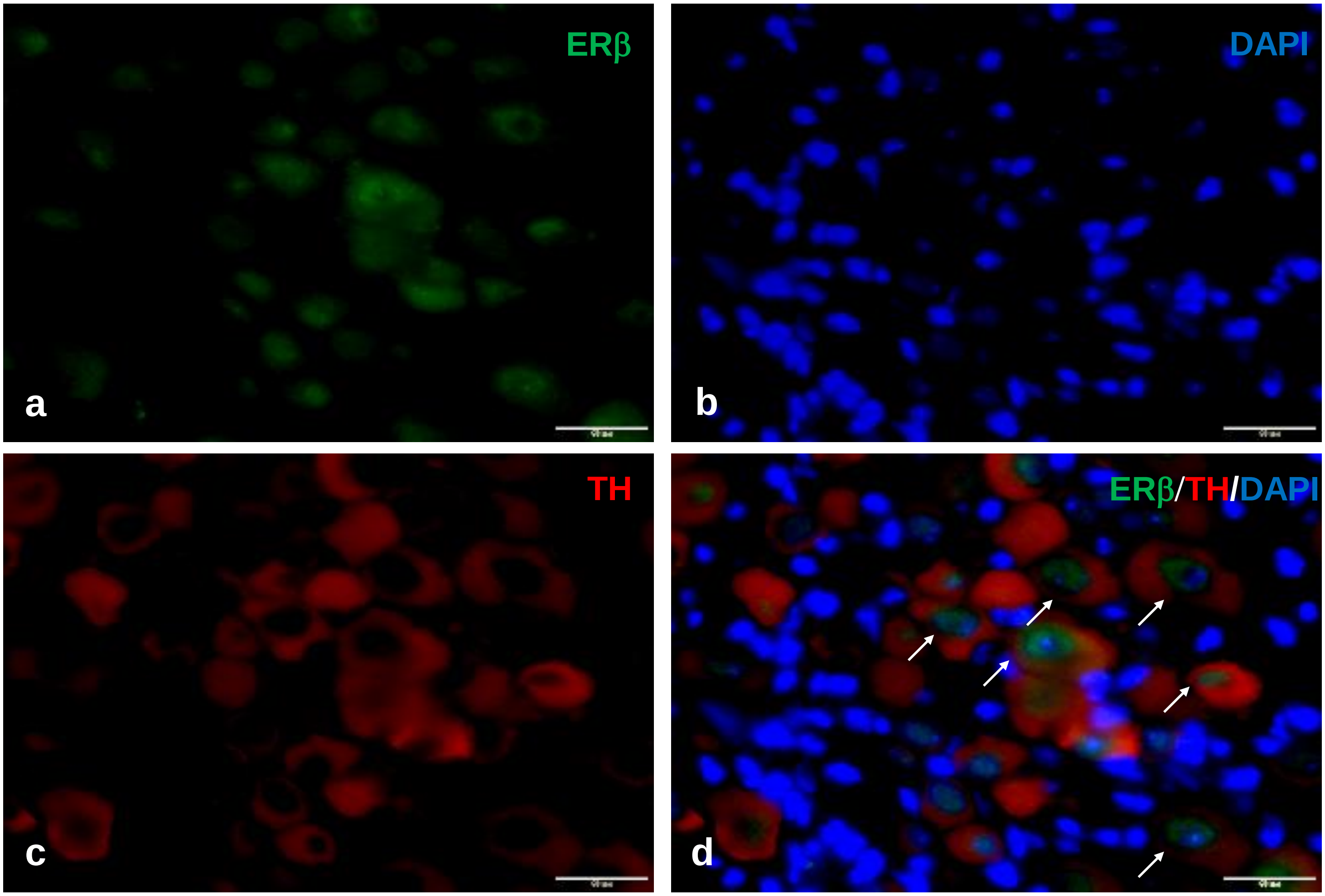

Supplementary Figure S3. Co-localization of ERβ with TH in the sympathetic ganglia (SG) of young female mice by immunofluorescence. ERβ staining appeared as green fluorescence (a), nuclei were marked with DAPI (b), whereas TH staining appeared as red fluorescence (c). Merged image (d) indicated that in the sympathetic ganglia of young female mice, ERβ was mostly expressed in nuclei (white arrows), and was rarely present in the cytoplasm indicated by TH expression.

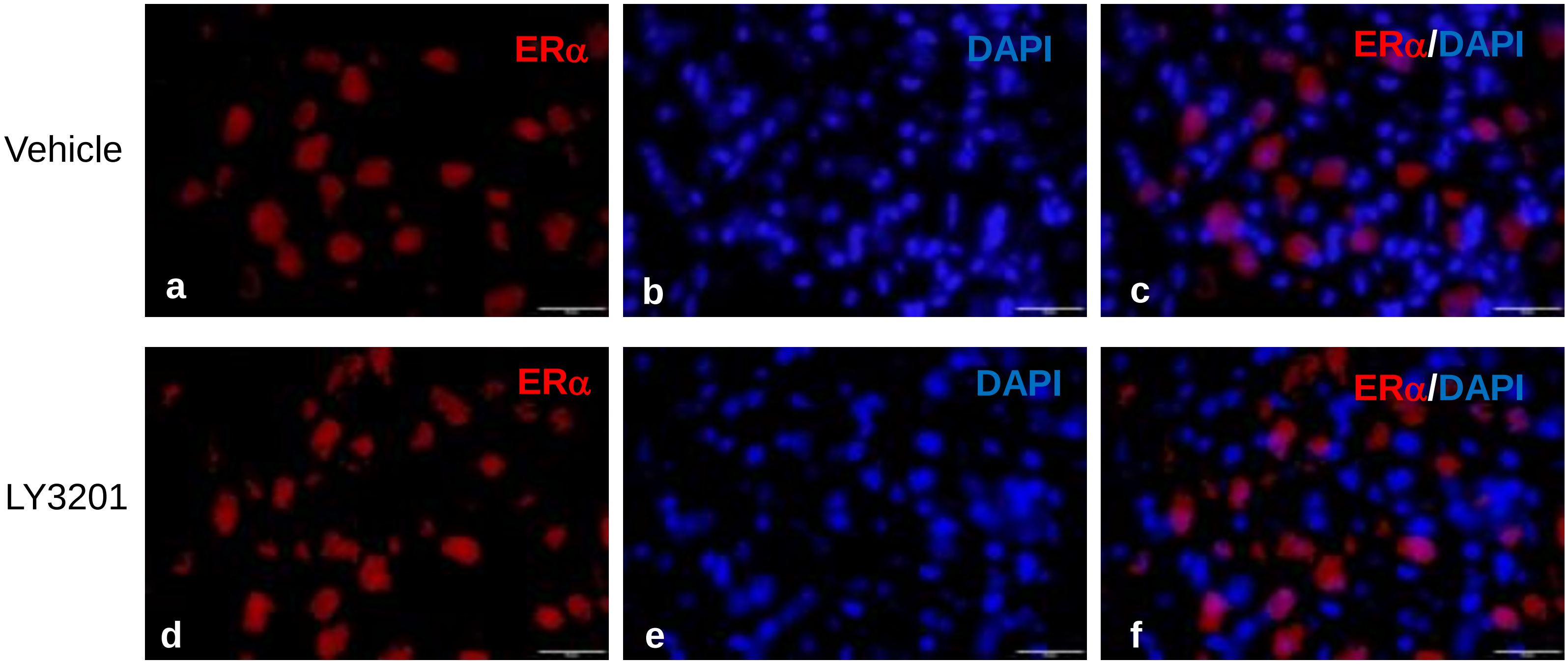

Supplementary Figure S4. SG ERα expression in both vehicle and LY3201-treated mice. Immunofluorescence results indicated that ERα was expressed in the nuclei of sympathetic neurons (c, f). No difference in ERα expression was found after 3 days' treatment with ERβ agonist (a, d). Scale bar, 50μm.

Supplementary Figure S4

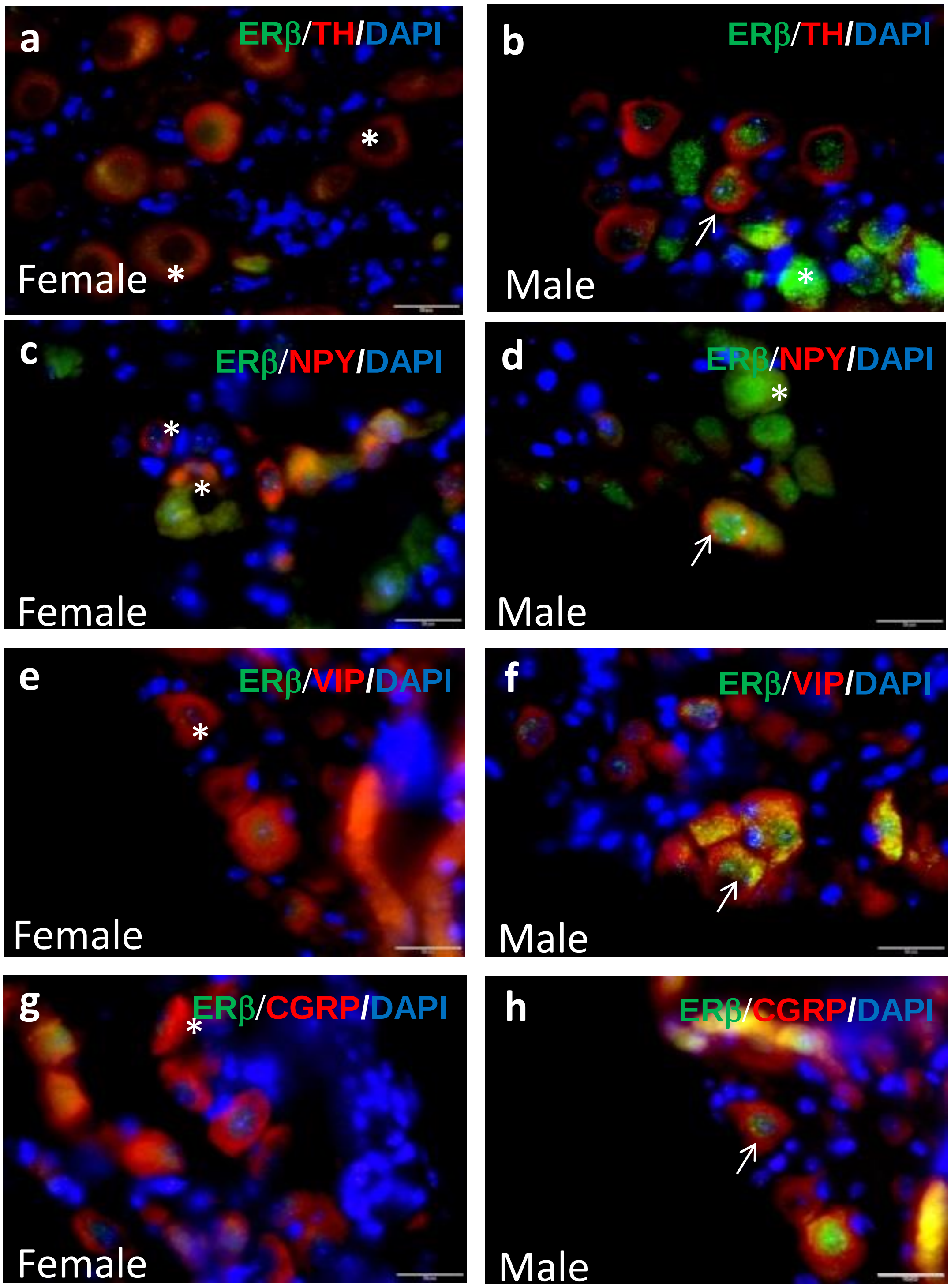

Supplementary Figure S5. Triple labeled immunostaining of anti-ERβ (green) and anti-TH (a and b, red)/NPY (c and d, red) /VIP (e and f, red) /CGRP (g and h, red) in 1-year-female (left panel) and 1-year-male (right panel) thoraco-lumbar sympathetic neurons. Asterisks indicate in old female mice, some TH-positive neurons lose nuclear ERβ signal. The white arrows indicate in the old male mice, ERβ is mainly expressed in nucleus. Scale bar=20 μm.

Supplementary Figure S5

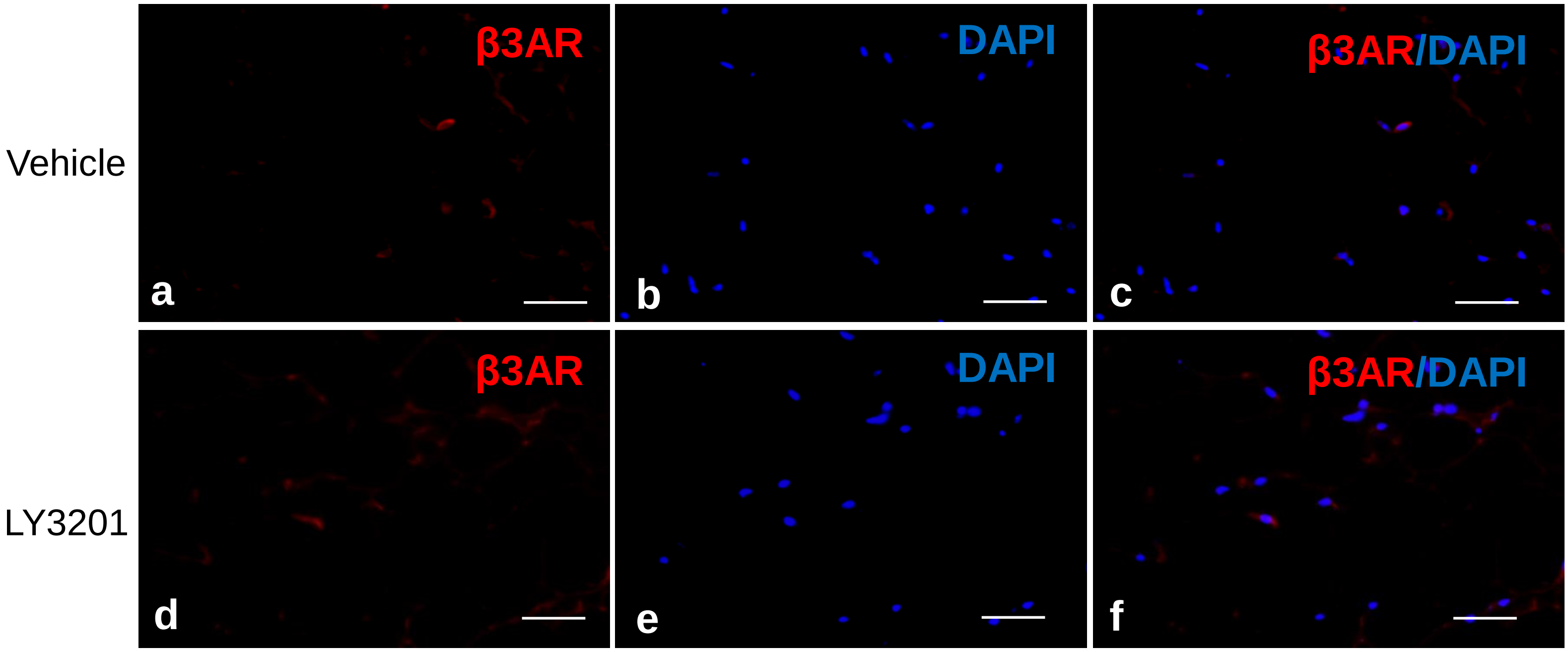

Supplementary Figure S6. Immunofluorescence results showed that LY3201 treatment failed to induce β3-adrenoceptor (B3AR) protein expression in 1-year-old male mice (Upper panel is vehicle group representative, lower panel is LY treated group representative).

Supplemental Table 1: Q-PCR primers

Genes	Forward (5'→3')	Reverse (5'→3')
UCP1	GATCCAAGGTGAAGGCCAGG	GTTGACAAGCTTTCTGTGG
Cidea	TGCTCTTCTGTATCGCCCAGT	GCCGTGTTAAGGAATCTGCTG
PGC1α	CGGAAATCATATCCAACCAG	TGAGAACCGCTAGCAAGTTTG
PGC1β	AGGTGTTCGGTGAGATTGTA	CCAGATGAGGGAAGGGAC
PPARγ	TGCAACCGTTACCCCATAGAA	TGCAACCGTTACCCCATAGAA
AP2	GCGTGGAATTCGATGAAATCA	CCCGCCATCTAGGGTTATGA
Tbx1	GGCAGGCAGACGAATGTTC	TTGTCATCTACGGGCACAAAG
Tmem26	ACCCTGTCATCCCACAGAG	TGTTTGGTGGAGTCCTAAGGTC
CD137	CGTGCAGAACTCCTGTGATAAC	GTCCACCTATGCTGGAGAAGG
Prdm16	CTTCTCCGAGATCCGAAACTTC	GATCTCAGGCCGTTTGTCCAT
pRb	CTGGCCTGTGCTCTTGAAGTT	CCACGGGAAGGACAAATCTGT
Foxc2	TCCATGGGAACCTTCTTCGA	GATCTCAAACTGAGCTGCGGATA
DIO2	ATGGGACTCCTCAGCGTAGA	GCACAGGCAAAGTCAAGAAG
Cyclin A2	GCCTTCACCATTCATGTGGAT	TTGCTCCGGGTAAAGAGACAG
Cyclin B1	AAGGTGCCTGTGTGTGAACC	GTCAGCCCCATCATCTGCG
PCNA	TTTGAGGCACGCCTGATCC	GGAGACGTGAGACGAGTCCAT
Ki67	ATCATTGACCGCTCCTTTAGGT	GCTCGCCTTGATGGTTCCT
C/EBPα	CAAGAACAGCAACGAGTACCG	GTCACTGGTCAACTCCAGCAC
C/EBPδ	CGACTTCAGCGCCTACATTGA	CTAGCGACAGACCCCACAC
